# Neuron type-specific optogenetic stimulation for differential stroke recovery in chronic capsular infarct

**DOI:** 10.1038/s12276-024-01253-8

**Published:** 2024-06-03

**Authors:** Ra Gyung Kim, Jongwook Cho, Ji-Young Park, Young Ro Kim, Min-Cheol Lee, Hyoung-Ihl Kim

**Affiliations:** 1 Department of Biomedical Science and Engineering, Gwangju Institute of Science and Technology, 123 Choemdangwagi-ro, Buk-gu, Gwangju, 61005 Republic of Korea; 2 Research Headquarter, Korea Brain Research Institute, 61 Cheomdan-ro, Dong-gu, Daegu, 41062 Republic of Korea; 3 Athinoula A. Martinos Center for Biomedical Imaging, Massachusetts General Hospital, Charlestown, MA 02129 USA; 4 Pathology Center, Seegene Medical Foundation, 320 Cheonho-Daero, Seongdong-gu, Seoul, 04805 Republic of Korea; 5 Department of Neurosurgery, Presbyterian Medical Center, 365 Seowon-ro, Wansan-gu, Jeonju-si, Jeollabuk-do 54987 Republic of Korea

**Keywords:** Stroke, Translational research

## Abstract

Cortical neuromodulation (CNM) is widely used to promote recovery after stroke. Despite the beneficial results of CNM, the roles played by different neuron types in the effects of current CNM techniques are unable to be differentiated. Our aim was to use selective optogenetic cortical stimulation to explore how different subpopulations of neuronal cells contribute to poststroke recovery. We transduced the sensory-parietal cortex (SPC) of rats with CamKII-ChR2 (pyramidal neurons), PV-ChR2 (parvalbumin-expressing inhibitory neurons), or hSyn-ChR2 (pan-neuronal population) before inducing photothrombotic capsular infarct lesions. We found that selective stimulation of inhibitory neurons resulted in significantly greater motor recovery than stimulation of excitatory neurons or the pan-neuronal population. Furthermore, 2-deoxy-2-[^18^F] fluoro-D-glucose microPET (FDG-microPET) imaging revealed a significant reduction in cortical diaschisis and activation of the corticostriatal neural circuit, which were correlated with behavioral recovery in the PV-ChR2 group. The spatial pattern of brain-derived neurotrophic factor (BDNF) expression was evident in the stimulated cortex and underlying cortico-subcortical circuit. Our results indicate that the plasticity of inhibitory neurons is crucial for functional recovery after capsular infarct. Modifying CNM parameters to potentiate the stimulation of inhibitory neurons could improve poststroke outcomes.

## Inhibitory neuron plasticity key to functional recovery post-stroke

Stroke, a major cause of long-term disability, has few treatment options. This research explores cortical neuromodulation, a method that changes brain activity, to improve motor functions and life quality for stroke patients. The study used optogenetic technology, which enables accurate stimulation of specific neuron types, to see how different neurons aid recovery after a stroke. They discovered that stimulating a particular neuron type, parvalbumin-expressing neurons, led to the most recovery. The research involved 8-week-old male rats, divided into three groups: control, sham, and optogenetic stimulation. They used a method called single pellet reaching task (SPRT) to assess the rats’ recovery after a stroke. The results showed that the group with optogenetic stimulation of parvalbumin-expressing neurons recovered the most. Moreover, they found that recovery circuit activation depends on the specific neurons stimulated.

This summary was initially drafted using artificial intelligence, then revised and fact-checked by the author.

## Introduction

Stroke is a major cause of long-term disability, yet treatment options for stroke patients with disabilities are limited. Many neuromodulation methods have been developed to enhance residual motor functions and improve the quality of life for stroke victims^1–4^. Among these techniques, cortical neuromodulation can be either invasive or noninvasive and has been widely used for poststroke recovery^5–11^. Although the mechanisms of poststroke recovery are not fully understood, there is a consensus that neuronal reorganization and synaptic plasticity are critical for promoting functional recovery from poststroke disability^12^. CNM modulates brain excitability, which may contribute to functional reorganization^13^. Enhanced brain excitability is believed to promote motor recovery, memory formation, and cortical learning by inducing long-term potentiation (LTP), a form of synaptic plasticity^9,11,14^. However, CNM modulates regulates activity over a large volume of brain tissue, making it difficult to predict the overall effects because of the summation across different networks and types of brain cells within the affected volume^15^. Furthermore, the roles of different neuronal populations in promoting recovery cannot be pinpointed using current CNM techniques^15,16^. Given that brain cells form unique neural circuits depending upon the neuronal type, it is necessary to understand how different types of neurons and their associated networks contribute to functional recovery after CNM.

Optogenetic technology enables cell type-specific stimulation of neuronal cells with millisecond precision^13^. Recently, poststroke optogenetic stimulation has gained widespread attention in the field of stroke research because of its potential for revealing the mechanisms of recovery after ischemia. Several studies have attempted to use optogenetic stimulation to rescue deficits caused by stroke or to define the role of local circuit modulation in enhancing recovery following ischemia^17–20^. Although these studies suggest that optogenetic modulation of the brain may indeed promote functional recovery from stroke, the role of different types of neurons in recovery and how brain networks respond to selective stimulation of specific neuron types remain unknown.

Previously, we showed that the capsular infarct model exhibits different pathophysiologies and recovery patterns^21,22^. A circumscribed capsular infarct is associated with retrograde Wallerian degeneration from the lesion in the internal capsule, advancing toward the ipsilesional motor and sensory cortices, resulting in cortical diaschisis^23^. Conventional motor cortex stimulation is not effective at promoting poststroke recovery, but cortical stimulation in the sensory-parietal cortex has been shown to promote successful recovery in a capsular infarct model^24^. Given that the neocortex contains a variety of neuronal types and actively participates in intricate neuronal network interactions^25^, questions have arisen regarding which specific neuronal cell types and their associated neural circuits play roles in capsular infarct recovery. Although a recent functional MRI study revealed an activated neural circuit in a cortical infarct model^20^, delineating the specific types of neuronal subpopulations and associated neural circuit mechanisms that drive poststroke recovery in capsular infarcts remains challenging.

In our current study, we employed the optogenetic technique to stimulate distinct neuronal types in a rat model of chronic capsular infarct, which manifests as persistent motor deficits^26,27^. Our findings indicate that selective optogenetic stimulation of different types of neurons in the cortex results in varying levels of poststroke recovery. Furthermore, micro-PET imaging showed that the activation of a recovery circuit depends on the specific types of neurons being stimulated. Overall, our study showed that selective stimulation of specific types of neurons and their associated circuits is critical for rescuing the poststroke deficit in capsular infarct. These observations could contribute to the adjustment of CNM parameters for individuals who have experienced stroke, providing an improved possibility of recovery for patients with chronic capsular infarcts.

## Materials and methods

### Experimental animals

Eight-week-old male Sprague‒Dawley rats (280–300 g) were used for the study. Rats were housed under a 12 h light–dark cycle with water provided ad libitum. All experiments were conducted in accordance with the Animal Research: Reporting of In Vivo Experiments (ARRIVE) guidelines. All experimental procedures were approved by the Gwangju Institute of Science and Technology Animal Care and Use Committee (GIST-2019–074).

All the animals underwent capsular infarct modeling and were observed to exhibit persistent motor deficits for 2 weeks. Then, the animals were divided into three main groups: the control group, the sham group and the optogenetic stimulation group. The control group (control: *n* = 8) did not receive virus injection but received optical stimulation through implanted optical fibers to rule out the possibility of prolonged optic stimulation-induced heat^28^. Animals in the sham group received an injection of one of three viral vectors with promoters for CamKII (CamKII-Sham: *n* = 7), parvalbumin (PV) (PV-Sham: *n* = 8), or hSyn (hSyn-Sham: *n* = 7) to eliminate potential comparison errors due to differences in viral vectors, but these animals did not receive optical stimulation. The animals in the optogenetic stimulation group were administered one of three viral vectors with different promoters: CamKII (CamKII-ChR2: *n* = 8), which targets pyramidal neurons; PV (PV-ChR2: *n* = 8), which targets PV-expressing neurons; or hSyn (hSyn-ChR2: *n* = 8), which targets pan-neuronal transduction. These animals were also subjected to optical stimulation.

### Behavioral task

The single pellet reaching task (SPRT) was used throughout the experiment for daily behavioral evaluation, as described in our previous study^26^. The SPRT was administered by an experimenter who was blinded to the group assignments. In each daily session, twenty pellets were provided, and the percentage of successful reaches was recorded. The SPRT evaluations were conducted simultaneously with the delivery of stimulation (Fig. 2a).

### Intracranial delivery of the viral vector

Rats were anesthetized with a mixture of ketamine hydrochloride (100 mg/kg) and xylazine (7 mg/kg). A small craniotomy was made, and a NanoFil syringe with a 33-gauge needle was stereotactically inserted into the ipsilesional sensory-parietal cortex at the following distances from bregma: AP = − 4.0 mm; ML = 3.1 mm; and DV = − 1 mm. A microsyringe controller (micro4, World Precision Instruments, FL, USA) was used to infuse 1 µl of one of the viral vectors—either AAV5-PV-ChR2(H134R)-eYFP, AAV5-CamKII-ChR2(H134R)-eYFP, or AAV5-hSyn-ChR2(H134R)-eYFP (KIST Vector Core, Korea), targeting parvalbumin-positive inhibitory neurons, excitatory neurons, or panneurons, respectively—in the optogenetic stimulation and sham groups. The needle was left in place for 10 min before withdrawal. The viral vectors were purchased from KIST Vector Core. The wound was sutured, and ketoprofen (2 mg/kg, i.m.) was administered for pain relief.

### Induction of photothrombotic capsular infarction and optical fiber implantation

One week after viral vector injection, a photothrombotic subcortical infarction was created in the posterior limb of the internal capsule (PLIC) contralateral to the preferred forelimb, as described in our previous studies^24,26,27,29^. A small craniotomy was made 2 mm posterior to the bregma and 3.1 mm lateral to the midline. An optical neural interface (ONI), made up of an optical fiber (core: 62.5 µm; outer diameter: 125 µm) housed in a 27-gauge steel cannula, was stereotactically inserted 7.8 mm deep into the PLIC. Following the infusion of Rose Bengal dye (20 mg/kg) through the tail vein, the PLIC was irradiated using the ONI with a green laser (532 nm, Changchun New Industries Optoelectronics Tech. Co., Ltd., Jilin, China) for 1.5 min. The laser light intensity at the tip of the fiber was 3.7 ± 0.2 mW. After withdrawal of the ONI, the optical fiber with a plastic ferrule was set in place, with the fiber tip located where the virus was previously infused. The fiber was secured to the skull with Metabond (C&B Metabond, Parkell, NY, USA) and dental cement. The wound was closed, and ketoprofen (2 mg/kg, i.m.) was administered.

### Optogenetic stimulation in the SPC

Based on our previous experiments, the SPC was identified as an optimal target for optogenetic manipulation in a capsular infarct model^24,30^. The implanted optical fiber was connected to a blue-light laser (Changchun, China), and a function generator (Master 9, A.M.P.I., Jerusalem, Israel) was used to control light-pulse delivery. The optogenetic stimulation group and the control group were subjected to two sessions of 1 h of stimulation daily for two weeks. Each session included pulses lasting 30 ms at a frequency of 10 Hz based on our previous studies^18,30–32^. The laser power was measured before and after each stimulation session and was consistently 1 mW at the fiber tip. Daily SPRT testing was conducted during one of the two daily stimulation sessions. The sham groups did not receive any light irradiation. Only rats with persistent motor impairment two weeks after subcortical infarction were subjected to optogenetic stimulation.

### Micro-PET/CT image acquisition and preprocessing

The animals underwent four micro-PET/CT scans: the initial scan was taken prior to the lesion (baseline), the second scan was acquired after the subcortical infarction (prestimulation, PS-1), and the final two scans were taken on the 7th (PS7) and 14th (PS14) days of stimulation. For the scan, an injection of ^18^F-FDG (100 mCi/100 g) was administered to the animals through the tail vein under brief isoflurane anesthesia, followed by a 30-min uptake period. During the uptake period, the optogenetic stimulation group and the control group received laser-light irradiation. Subsequently, static PET scans (25 min) and attenuation correction computerized tomography (5 min) were performed using an Inveon PET/CT scanner (Siemens Medical Solutions, TN, USA) under anesthesia. Vital signs such as respiration, heart rate, and body temperature were monitored throughout the scan (BioVet System, m2m Imaging Corp., OH, USA). After scanning, the image data underwent attenuation correction and reconstruction using the iterative OSEM3D/MAP algorithm.

Analysis of Functional NeuroImages (AFNI, National Institutes of Health, MD, USA)^33^, a software package for the analysis and visualization of three-dimensional functional images, was used for microPET image analysis. All images were coregistered to an MRI template^34^ and normalized using an intensity-scaling approach. Finally, the images were spatially smoothed using an isotropic Gaussian kernel with 1.2 mm full width at half maximum.

### Histological analysis

After the microPET/CT scan was completed, all rats in each group were sacrificed for histological evaluation. Under ketamine anesthesia (100 mg/kg body weight), the animals were perfused with 0.9% saline, followed by 4% paraformaldehyde (PFA). The brains were postfixed overnight in 4% PFA and transferred to 30% sucrose to ensure cryoprotection. Brain blocks embedded in OCT compound (Cell Path, Newtown, UK) were cut on a freezing microtome (Cryocut 3000, Leica Biosystems, Nussloch, Germany) at 40 µm thickness.

To confirm viral expression in the target neuron types, GFP staining (1:1000, A11122, Molecular Probes) was performed. Furthermore, immunohistochemistry was performed using specific antibodies for each neuron type: anti-CamKII (1:200, AB22609, Abcam) for excitatory neurons, anti-parvalbumin (1:1000, 195004, Synaptic Systems) for inhibitory neurons and anti-NeuN (1:200, MAB377, Chemicon) for pan-neuronal cells.

To detect BDNF-positive cells, anti-BDNF (1:300, 25699-1-AP, ProteinTech) and anti-NeuN antibodies were used. We quantified the population of BDNF-positive cells among the NeuN-positive cells by counting the number of cells positive for both markers in the regions that showed significant activation in the FDG-microPET study. These regions included the ipsilesional motor cortex, bilateral striatum, and thalamus. From each of these regions, we selected three regions of interest (ROIs) measuring 200 μm × 200 μm for analysis.

### Statistical analysis

The longitudinal microPET data were analyzed using a group-level linear mixed-effect model through the 3dLME program within the AFNI. To assess changes in cortical diaschisis volume, we contrasted prelesional (PL) microPET images with postlesional stimulation (PS −1, PS7 and PS14) images. Statistical maps were thresholded at the significance level (*p* < 0.001, false discovery rate *q* < 0.05). An ROI mask was created based on statistically thresholded maps of PS −1 with a threshold of *p* < 0.001 for each group to measure changes in normalized mean activity (NMA) in the cortical diaschisis area. Furthermore, image analysis was performed to probe the effect of neuron-specific optogenetic stimulation by comparing prestimulation images (PS −1) to images after stimulation (PS7 and PS14). These maps were thresholded at the significance level (*p* < 0.01). ROIs were defined manually in the activated regions for each individual group.

The data were analyzed in Prism 7 (GraphPad Software Inc., CA, USA). Infarct volume data were analyzed with one-way ANOVA with Tukey’s multiple comparisons test. Longitudinal changes in cortical diaschisis volume and the NMA and SPRT data were analyzed via repeated-measures two-way ANOVA with Tukey’s multiple comparisons test. In addition, percentage changes in regional NMA data were analyzed using Brown-Forsythe one-way ANOVA with Dunnett’s T3 multiple comparison test. BDNF data were analyzed with one-way ANOVA with Tukey’s multiple comparisons test. Linear regression (*p* < 0.05) was used to measure the correlations between the NMA in the cortical diaschisis region and the SPRT scores. All the data are presented as the mean ± standard error of the mean (S.E.M.). The significance level is indicated by asterisks (*p < 0.05, **p < 0.01, ***p < 0.001; ns not significant).

## Results

### Capsular infarct model and viral transduction of specific neuron types

**Fig. 1:**
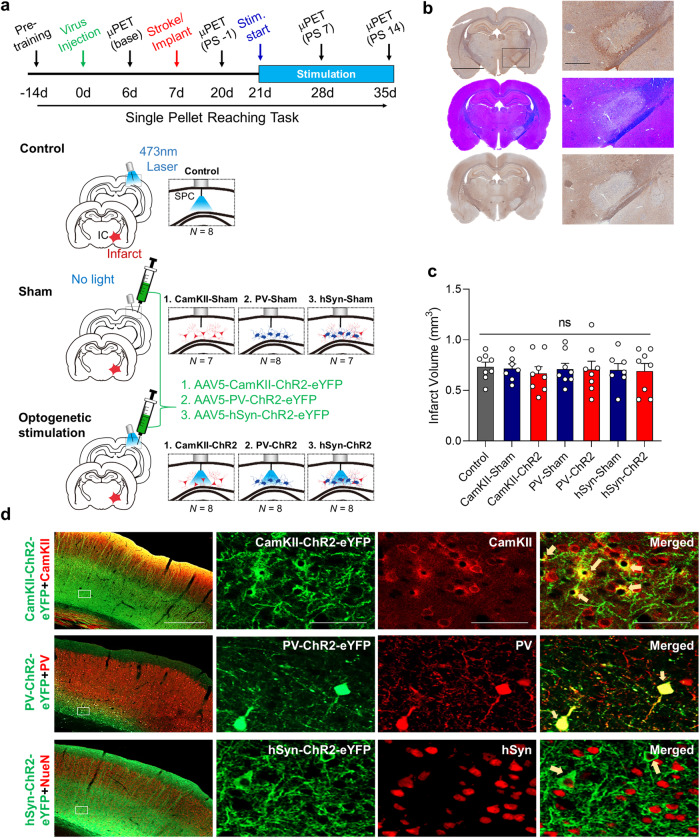
Optogenetic cortical stimulation in a rodent model of capsular infarct. **a** Schematic drawing of the experimental design and animal grouping. The control group underwent photothrombotic infarct lesioning in the posterior limb of the internal capsule (red star) and received optical stimulation (marked in blue) but did not receive a viral injection (green syringe). The sham groups underwent infarct lesioning, viral injection, and implantation of the optical fiber but did not receive optical stimulation. The animals in the optogenetic stimulation groups (*n* = 42) underwent infarct lesioning viral injection and optical stimulation. **b** Capsular infarcts stained for GFAP (top), Luxol-fast blue (middle), and neurofilaments (bottom). Scale bars: 4 mm (left) and 100 µm (right). **c** Bar graph showing the infarct sizes in all groups. There were no significant differences in infarct size among the groups (one-way ANOVA *P* < 0.05). The data are presented as mean ± S.E.M. **d** Representative confocal images demonstrating the coexpression of eYFP and cells positive for CamKII, PV, or hSyn (white arrows). Scale bars: 1 mm (left column) and 200 µm (right 3 columns).

We used a chronic capsular infarct rat model, as previously described, because this model produces a chronic motor deficit from which the animals do not recover naturally^26^. Furthermore, poststroke recovery can be accurately evaluated with postrehabilitation behavioral testing and functional imaging^27,35^. The primary goal of our study was to establish the effects of optogenetic cortical stimulation (OCS) on specific types of neurons. For this purpose, rats in the stimulation group were randomly divided into three subgroups after training on the SPRT for viral injection in the sensory-parietal cortex (SPC): CamKII-ChR2 for stimulating pyramidal neurons, PV-ChR2 for stimulating PV-expressing neurons, or hSyn-ChR2 for pan-neuronal stimulation (Fig. 1a). One week after viral injection, all animals underwent photothrombotic capsular infarction of the PLIC contralateral to the preferred forelimb (Fig. 1b). Upon quantification of the lesion, the infarct volume did not significantly differ among the groups that received different viral injections (Fig. 1c). For neuron type-dependent ChR2 expression, histological examination confirmed the colocalization of ChR2-eYFP to CamKII-positive cells in the CamKII-ChR2 group, to PV-positive cells in the PV-ChR2 group, and to NeuN-positive cells in the hSyn-ChR2 group (Fig. 1d).

### Behavioral recovery varies with the specific neuron type stimulated

**Fig. 2:**
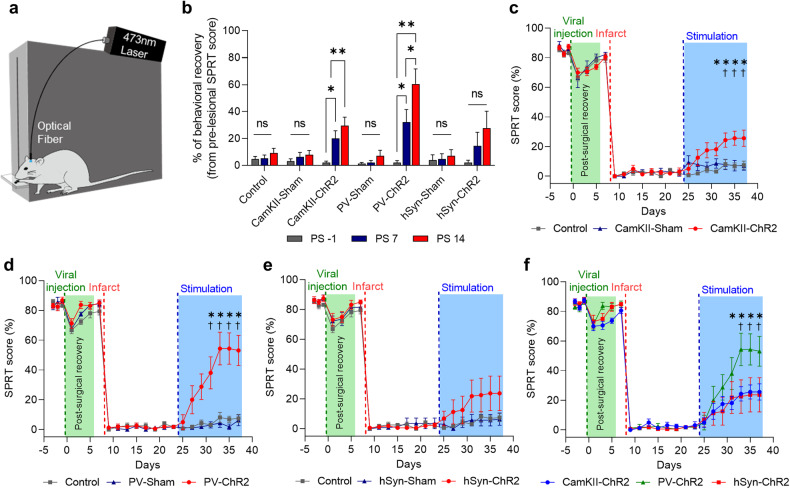
Optogenetic stimulation of PV neurons leads to the greatest motor recovery. **a** Schematic diagram of the daily SPRT with concurrent optogenetic stimulation. **b** Behavioral recovery relative to prelesional SPRT scores across the experimental groups (repeated-measures two-way ANOVA with Tukey’s multiple comparisons test, F (12, 94) = 5.009, *p* < 0.0001). **c**–**e** Behavioral performance depends on the type of optogenetic cortical stimulation (repeated-measures two-way ANOVA with Tukey’s multiple comparisons test; C, F (42, 420) = 2.943, *p* < 0.0001; D, F (42, 441) = 10.16, *p* < 0.0001; E, F (42, 420) = 1.372, *p* = 0.0665; *ChR2 vs. Control; ^†^ChR2 vs. Sham). **f** Comparison of daily SPRT scores among the optogenetic stimulation groups (repeated-measures two-way ANOVA with Tukey’s multiple comparisons, F (42, 441) = 2.244, *p* < 0.0001; *CamKII-ChR2 vs. PV-ChR2; ^†^hSyn-ChR2 vs. PV-ChR2). The blue shading indicates the period of optogenetic stimulation. All the data are presented as mean ± S.E.M. *p < 0.05, ^†^*p* < 0.05, **p < 0.01; ns not significant.

We applied OCS to the SPC based on our previous observation that stimulation of either the motor or premotor cortex did not yield beneficial recovery effects in capsular infarction^24^. We used daily SPRT performance to evaluate poststroke recovery (Fig. 2a). After confirming that the animals showed persistent motor deficits ( >3 weeks) after capsular infarct lesioning, they received daily OCS two times a day for 2 weeks.

The OCS stimulation groups with different ChR2 expressions showed neuron type-dependent improvements in SPRT performance (Fig. 2). The PV-ChR2 group showed significant recovery, achieving SPRT scores up to 60.5% of their scores prior to lesion induction (Fig. 2b). This was followed by the CamKII-ChR2 group, whose recovery was 29.5%, and then the hSyn-ChR2 group, whose recovery was 27.8% (Fig. 2b). Compared with the control and sham groups, the PV-ChR2 and CamKII-ChR2 groups exhibited significant motor improvement, while the hSyn-ChR2 group did not show significant recovery during the observation period (Fig. 2c–e). Interestingly, the PV-ChR2 group showed the most significant recovery among the 3 CNM groups (Fig. 2f). These findings indicate the potential of OCS to enhance poststroke recovery. Notably, the stimulation of PV cells yielded the most significant motor improvement. This finding challenges the traditional belief that pyramidal neurons are the primary players in cortical excitability during cortical stimulation and consequent poststroke recovery^36,37^. In contrast, the OCS of PV-expressing neurons contributed the most to the enhancement of poststroke recovery. This result implies that cell type specificity should be taken into consideration, and further study is required to examine the influence of cell type-specific stimulation on functional recovery and to elucidate the mechanism of cell type-specific effects on behavior and circuit changes.

### Neuron type-specific stimulation can improve cortical excitability

**Fig. 3:**
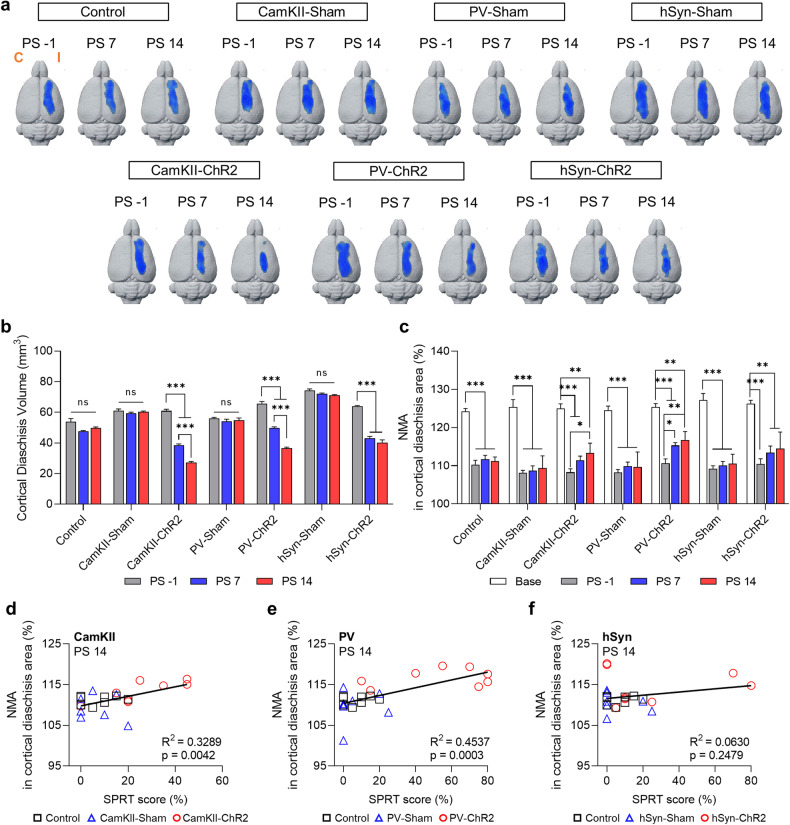
Reductions in cortical diaschisis correlate with behavioral outcomes. **a** Longitudinal changes in cortical diaschisis in all groups over time. Blue indicates regions with a significant reduction in regional glucose metabolism (3dLME in AFNI, *p* = 0.001, false discovery rate *q* < 0.05) The microPET image analysis was performed between prelesional images (PL) and postlesional images (PS -1, PS7 and PS14) to assess diaschisis. C, contralesional; I, ipsilesional. **b** Time-dependent changes in the volume of cortical diaschisis (repeated-measures two-way ANOVA with Tukey’s multiple comparisons, F (12, 94) = 62.08, *p* < 0.0001). **c** Time-dependent change in normalized mean activity (NMA) in the diaschisis region (repeated-measures two-way ANOVA with Tukey’s multiple comparisons, F (18, 141) = 2.329, *p* = 0.0031). **d**–**f** Linear regression between the NMA in the cortical diaschisis region and the SPRT scores for each cell-specific stimulation group at PS 14. (D, F (1, 21) = 10.29; E, F (1, 22) = 18.27; F, F (1, 21) = 1.413). All the data are shown as mean ± S.E.M. *p < 0.05, **p < 0.01, ***p < 0.001; ns not significant.

Diaschisis is defined by depression of regional brain metabolism remote from the primary brain injury, and this phenomenon is a consistent finding in a capsular infarct model^22^. The occurrence of diaschisis is closely associated with clinical symptoms and recovery from stroke, as reported in our previous studies^24,27,35^. To monitor the time-dependent changes in diaschisis (i.e., functional reorganization of brain regions) upon OCS, we performed longitudinal FDG-microPET imaging before infarct lesioning (PL), before stimulation (PS −1), and on the 7th (PS7) and 14th (PS14) days of OCS. After the generation of the PLIC infarct lesion, all groups exhibited cortical diaschisis in the ipsilesional sensorimotor cortex. Notably, OCS significantly reduced the volume of diaschisis between PS7 and PS14 in the PV-ChR2 and CamKII-ChR2 groups (Fig. 3a, b). However, there was no significant reduction in the diaschisis volume between PS7 and PS14 in the hSyn-ChR2 group. The control group showed an initial reduction in normalized mean activity (NMA), with no significant change observed between PS7 and PS14. However, the PV-ChR2 and CamKII-ChR2 groups exhibited significant restoration of NMA in the area of diaschisis by PS14 (Fig. 3c). The NMA in the cortical diaschisis area was significantly correlated with behavioral improvements in the CamKII-ChR2 and PV-ChR2 groups, while no correlation was detected in the hSyn-ChR2 group (Fig. 3d–f). Taken together, our results show that the selective stimulation of PV-expressing inhibitory neurons results in a significant correlation between a reduction in diaschisis and behavioral recovery.

### Neuron type-specific stimulation induces the activation of distinct neural circuits

**Fig. 4:**
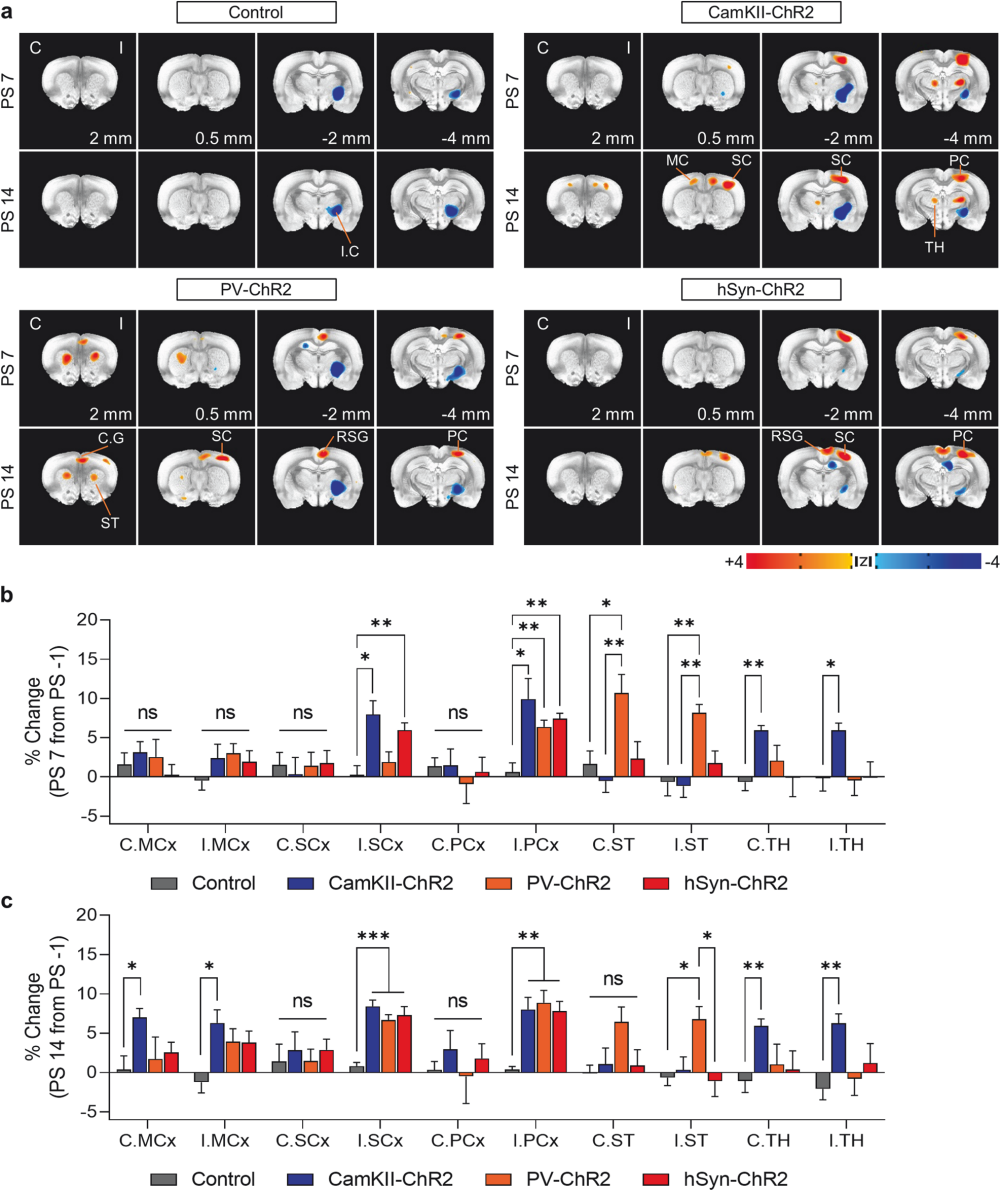
Optogenetic stimulation of different neuron types produces different patterns of activation in FDG-microPET images. **a** FDG-microPET images showing different activation patterns in each OCS group (3dLME in AFNI, *p* < 0.01). MicroPET image analysis was performed between prestimulation images (PS -1) and stimulation images (PS7 and PS14) to identify the stimulation effect. **b, c** Changes in regional NMA (% change from PS -1) in the control and OCS groups at PS7 and PS14 (Brown-Forsythe one-way ANOVA with Dunnett T3 multiple comparison test). See also Supplementary Fig. 1. All the data are presented as mean ± S.E.M. *p < 0.05, **p < 0.01, ***p < 0.001. C contralesional, I ipsilesional, I.C. internal capsule, MC motor cortex, SC sensory cortex, PC parietal cortex, C.G. cingulate gyrus, TH thalamus, ST striatum, RSG retrosplenial cortex.

CNM is thought to enhance motor recovery after chronic stroke by inducing efficient motor–skill learning though the reorganization of neural substrates recruited during cortical stimulation^13,38^. Studies exploring the neural substrates of motor-skill learning after stroke have revealed increased activity in specific structures, such as the striatum, premotor cortex, and motor cortex, after CNM^13,24,39^. We performed longitudinal FDG-microPET to investigate how the recruitment of neural substrates by OCS varies depending on the specific types of neurons being stimulated. We performed microPET scans before stimulation (PS −1) and on the 7th (PS7) and 14th (PS14) days of OCS to assess longitudinal changes in regional glucose metabolism (rGM) (Fig. 4 and Supplementary Fig. 1).

All groups showed a marked decrease in rGM at the site of the infarct lesion. OCS produced an increase in the rGM in the ipsilateral parietal cortex (the virus expression and stimulation site) in the CamKII-ChR2, PV-ChR2, and hSyn-ChR2 groups. The PV-ChR2 group, which exhibited the greatest behavioral recovery, had markedly increased rGM in both cortical and subcortical structures encompassing the ipsilateral sensory and parietal cortices and bilateral striata, indicating the activation of the corticostriatal circuit. This activation in the striatum peaked at PS7 and decreased by PS14 (Fig. 4). The CamKII-ChR2 group exhibited activation of the corticothalamic circuit only, and the degree of thalamic activation was relatively small. Conversely, the hSyn-ChR2 group showed a persistent increase in rGM in cortical structures, including the ipsilateral sensory and parietal cortices, but there was no activation of subcortical structures. These findings underscore the importance of stimulating neural circuits involving both cortical and subcortical structures to improve functional recovery in capsular infarcts. Taken together, these findings indicate that distinct neuron types trigger the activation of different neural circuits. The PV-ChR2 group exhibited activation of the corticostriatal circuit and the most successful recovery, whereas the CamKII-ChR2 group showed activation of the corticothalamic circuit and less successful recovery. The hSyn-ChR2 group showed persistent activation of cortical structures without subcortical activation and motor improvements similar to those of the CamKII-ChR2 group. These findings are consistent with previous functional imaging studies of motor learning^40,41^. Consequently, enhancing motor recovery after capsular infarct is dependent on activating the corticostriatal circuit, which encompasses both the cortex and striatum.

### BDNF expression is correlated with neural network activation

**Fig. 5:**
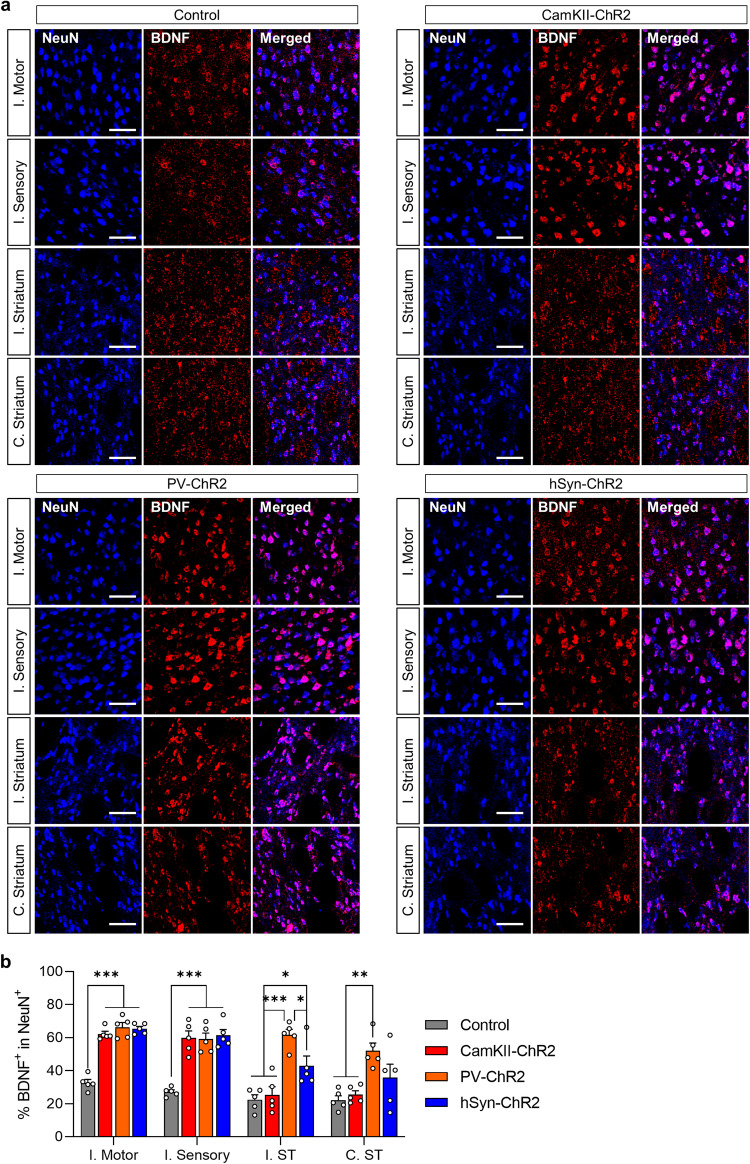
Optogenetic neuronal stimulation increases BDNF expression. **a** Representative images of BDNF^+^ and NeuN^+^ cells in the control and OCS groups. Scale bars: 50 µm. **b** The percentage of BDNF^+^ population within NeuN^+^ cells in four different brain regions (one-way ANOVA with Tukey’s multiple comparisons). All the data are presented as mean ± S.E.M. *p < 0.05, **p < 0.01, ***p < 0.001. See also Supplementary Fig. 2.

The functional imaging activation patterns described above reflect neural excitability. One of the functional consequences of this hyperexcitability is the facilitation of activity-dependent plastic changes^39,42^. Given the diverse impacts of neuron type-specific OCS on motor recovery, it was important to explore the underlying mechanism that might account for these differences. We investigated brain-derived neurotrophic factor (BDNF), a pivotal molecular marker, to gain insight into plastic changes induced by OCS. Compared with those in the control and sham groups (Fig. 5 and Supplementary Fig. 2), BDNF-positive cells in the ipsilateral motor and sensory cortices were significantly greater in all OCS groups. The PV-ChR2 group showed a significant increase in BDNF expression in cortical structures and subcortical structures, including the ipsilateral motor and sensory cortices and bilateral striata. This finding underscores the broad activation of neural circuits following PV neuron-specific OCS. CamKII-ChR2 primarily demonstrated enhanced BDNF expression in cortical structures only. The hSyn-ChR2 group exhibited a uniform increase in BDNF expression in most of the sampled regions; however, BDNF expression was significantly lower in the hSyn-ChR2 group than in the PV-ChR2 group. Overall, the expression in the PV-ChR2 group was more prominent than that in the other groups (Fig. 5), which potentially accounts for the better motor recovery in the PV-ChR2 group. One intriguing observation was the inconsistency between BDNF expression and FDG-microPET activation areas. While both tools are indicative of neural activity and subsequent plasticity, they do not always align perfectly. This discrepancy is likely caused by the target of the findings: FDG-microPET captures real-time metabolic activity reflecting immediate neural excitability, whereas BDNF expression represents long-term plastic changes induced by OCS. Therefore, the increase in BDNF expression was not consistent with the activated area observed in microPET images. It is hypothesized that areas of augmented metabolic activity are likely undergoing significant plastic remodeling, leading to motor recovery.

## Discussion

CNM is one of the most commonly used neuromodulation techniques for chronic stroke patients^6^. In most clinical trials, CNM is assumed to either directly or indirectly activate corticospinal fibers, which originate from pyramidal neurons in the cerebral cortex^43^. In fact, cortical stimulation affects all types of neurons located in the region of stimulation, and the overall effect of stimulation is the result of interactions among these neurons^44^. In this study, we performed OCS to explore how different subpopulations of neuronal cells contribute to poststroke recovery. The results described here demonstrate that the modulation of PV-expressing neurons is the most important in promoting poststroke recovery after subcortical capsular infarct. Importantly, the PV-ChR2 group exhibited activation of the corticostriatal circuit, which is critical for promoting functional recovery in capsular infarcts. The PV-ChR2 group also exhibited significantly greater expression of BDNF, which is related to plasticity. In addition, the PV-ChR2 group exhibited a significant reduction in diaschisis, which was correlated with behavioral outcomes. However, the selective stimulation of pyramidal neurons or the pan-neuronal population did not significantly contribute to recovery. This study suggested that the determination of cortical stimulation parameters may benefit from modifications directed toward activating PV-expressing neurons rather than random stimulation of all neuronal cells in the capsular infarct.

Recently, a number of studies have applied optogenetic stimulation of the ipsilesional and contralesional motor cortex, striatum, thalamus, or cerebellum via light-sensitive channels such as channelrhodopsin-2 (ChR2), which are targeted to pyramidal or pan-neuronal cell populations in animal stroke models^17–19,30,45^. However, most studies have focused on excitatory or pan-neuronal targets, based on the general concepts that excitatory pyramidal neurons form the vast majority of cortical neurons and that plasticity at pyramidal neuron synapses is believed to make the greatest contribution to overall cortical plasticity^46^. However, cortical stimulation alters synaptic efficacy in excitatory and inhibitory circuits, which are critical to synaptic plasticity^47–49^. In this study, we targeted pyramidal neurons, inhibitory neurons and pan-neuronal cells to compare the differential effect of OCS by varying the targets in capsular infarct. Notably, the stimulation of PV-expressing neurons resulted in the most significant recovery, suggesting that stimulation of the inhibitory neuronal population is the ideal target for CNM, if possible. It has been reported that the facilitation of inhibitory synapses is around twice as strong as that of excitatory synapses^50^. Recent reports have also highlighted the importance of activity-dependent short- and long-term plasticity at GABAergic synapses^51,52^. These reports support the vital role of inhibitory synapses in inducing synaptic plasticity associated with behavioral recovery.

The cortical sites of stimulation are the key factors for stroke recovery in CNM. The cortex is connected with the underlying white matter, forming cortico-cortical and cortico-subcortical connections. Brian stimulation induces the synchronization of firing through these networks^53^. It has been shown that TMS-related signal propagation in the brain is highly associated with structural networks rather than with functional networks^54^. Therefore, cortical stimulation is likely to induce different patterns of functional reorganization in the cortex and activation of different neural circuits depending on the type of stroke model. In this study, we used a capsular infarct model in which cortical damage is absent. In our previous study, the sensory parietal cortex was the most effective site of stimulation, whereas motor or premotor cortex stimulation did not contribute significantly to functional recovery^24^. Our microPET study revealed that the corticostriatal circuit is the most relevant network. The activation of the corticostriatal circuit is expected based on the anatomical network in which GABAergic neurons send long-range projections, such as those in the striatum or globus pallidus, and interneuron populations in the cortex^25^. The corticothalamic circuit was activated in the CamKII-ChR2 group, but could not achieve significant motor recovery. Additionally, the hSyn-ChR2 group, in which the cortex was activated without subcortical involvement, showed no significant motor recovery. These results contrast with those of other reports emphasizing the contribution of corticothalamic circuits to stroke recovery^20^. However, this discrepancy stems from differences in the animal stroke model, the site of stimulation and the type of neuronal population stimulated. Therefore, cortical stimulation strategies cannot be generalized to all types of stroke. We need to consider the different and individualized stimulation options depending on the site of infarction (cortical versus subcortical), type of stroke (gray versus white matter stroke), and stimulation target of the neuronal population or circuit.

Previously, we showed that subcortical capsular infarct induces Wallerian degeneration from the site at which the lesion reaches the cortical level^23^. We also demonstrated that GABA-synthesizing reactive astrocytes in cortical areas cause tonic inhibition of neighboring neurons, leading to the development of diaschisis^21^. Therefore, the reversal of diaschisis is an important contributing factor in poststroke deficits in capsular infarcts. In this study, diaschisis was reduced with OCS in all the groups regardless of the neuronal cell type. However, not all the groups showed a beneficial effect on functional recovery. Significant motor recovery was only observed in the PV-ChR2 group. Many trials have measured GABA levels, but the results are controversial^55^. We found that two types of GABA existed in the PV-ChR2 group: GABA released from reactive astrocytes after Wallerian degeneration in the cortex and neuronal GABA released from PV-expressing neurons in a capsular infarct model^21^. Astrocytic GABA seems to perturb neuronal function in the ipsilesional cortex following capsular infarct, as mentioned before, whereas neuronal GABA is hypothesized to be involved in poststroke neuronal plasticity mediated by Hebbian and homeostatic plasticity mechanisms^56,57^. GABAergic modulation is reportedly important for shaping spike-timing-dependent plasticity and operates as a Hebbian/anti-Hebbian switch^58,59^. Additionally, it is also important to balance the synaptic environment between excitatory and inhibitory inputs to maintain homeostatic plasticity. This study supports the hypothesis that GABAergic modulation is required to drive inhibition, which strengthens and retains properly wired connections in chronic stroke^55^.

In clinical practice, noninvasive brain stimulation (NIBS), including transcranial magnetic stimulation (TMS), transcranial direct current or alternating current (tDCS or tACS), is commonly utilized in stroke survivors. A recent report confirmed that NIBS is effective in facilitating motor recovery^60^. However, these techniques have limitations in selectively stimulating specific neuronal populations. However, Mahmud et al. showed that adjusting the wave parameters may provide selective enhancement or suppression of the excitatory/inhibitory ratio^61^. TMS also has the ability to drive GABA receptor activity using paired pulses with different interstimulus intervals or low-frequency stimulation^62,63^. Therefore, the development of optimal NIBS parameters that drive inhibitory neuronal stimulation needs to be pursued.

In this study, we used an optogenetic technique to selectively stimulate excitatory, inhibitory or pan-neuronal populations for functional recovery in an animal model of subcortical infarction, allowing us to elucidate the recovery mechanism. Selective stimulation of PV-expressing neurons resulted in the most significant recovery. We also found that cortical activation and the underlying corticostriatal circuit contribute the most to poststroke neural plasticity, leading to recovery. Our results suggest that neuron-type specificity should be taken into consideration in facilitating functional recovery and that modifying CNM parameters will likely be necessary for refining future strategies for treating capsular infarct.

### Supplementary information

Supplementary information

## Data Availability

All relevant data are available from the authors upon reasonable request.
