# Supplementary material for: Neuron type-specific optogenetic stimulation for differential stroke recovery in chronic capsular infarct
Source: Exp Mol Med. 2024 Jun 3;56(6):1439–49. doi: 10.1038/s12276-024-01253-8 (PMC11263592; doi:10.1038/s12276-024-01253-8)
Supplement: Supplementary file 1 — Supplementary information [file 12276_2024_1253_MOESM1_ESM.pdf]

Supplementary information

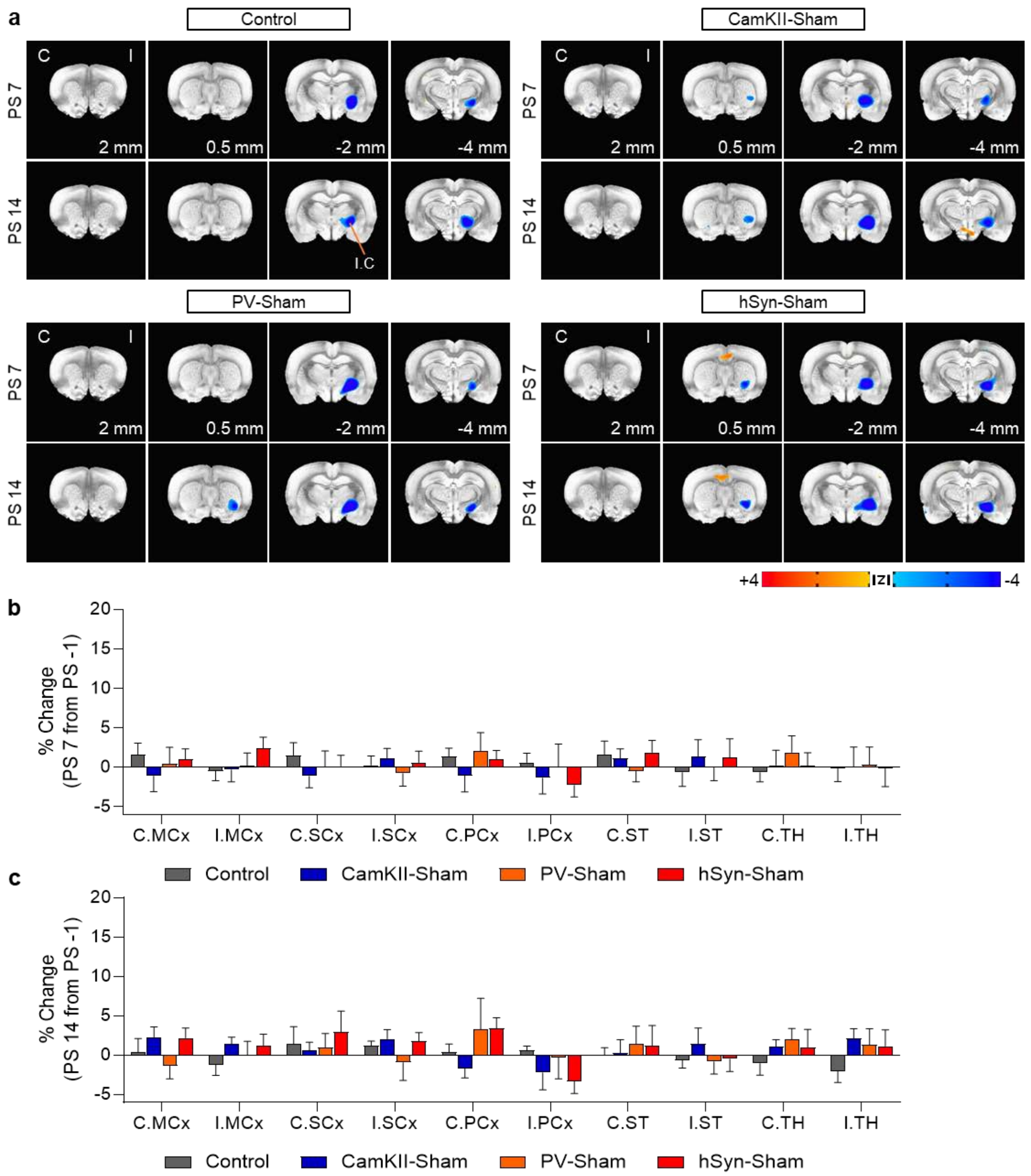

Supplementary Fig. 1. FDG-microPET images in the control and sham-stimulation groups.

(a) FDG-microPET imaging shows activation patterns in each neuronal type, sham-stimulation group (3dLME in AFNI, $p < 0.01$). The microPET image analysis was performed between pre-stimulation images (PS -1) and stimulation images (PS7 and PS14). **(b-c)** Changes of regional NMA (% change from the PS -1) in the control and sham-stimulation groups at PS 7 and PS 14. (Brown-Forsythe one-way ANOVA with Dunnett T3 multiple comparison test). All Data represent the mean ± S.E.M. C, Contralesional; I, Ipsilesional; I.C., Internal capsule.

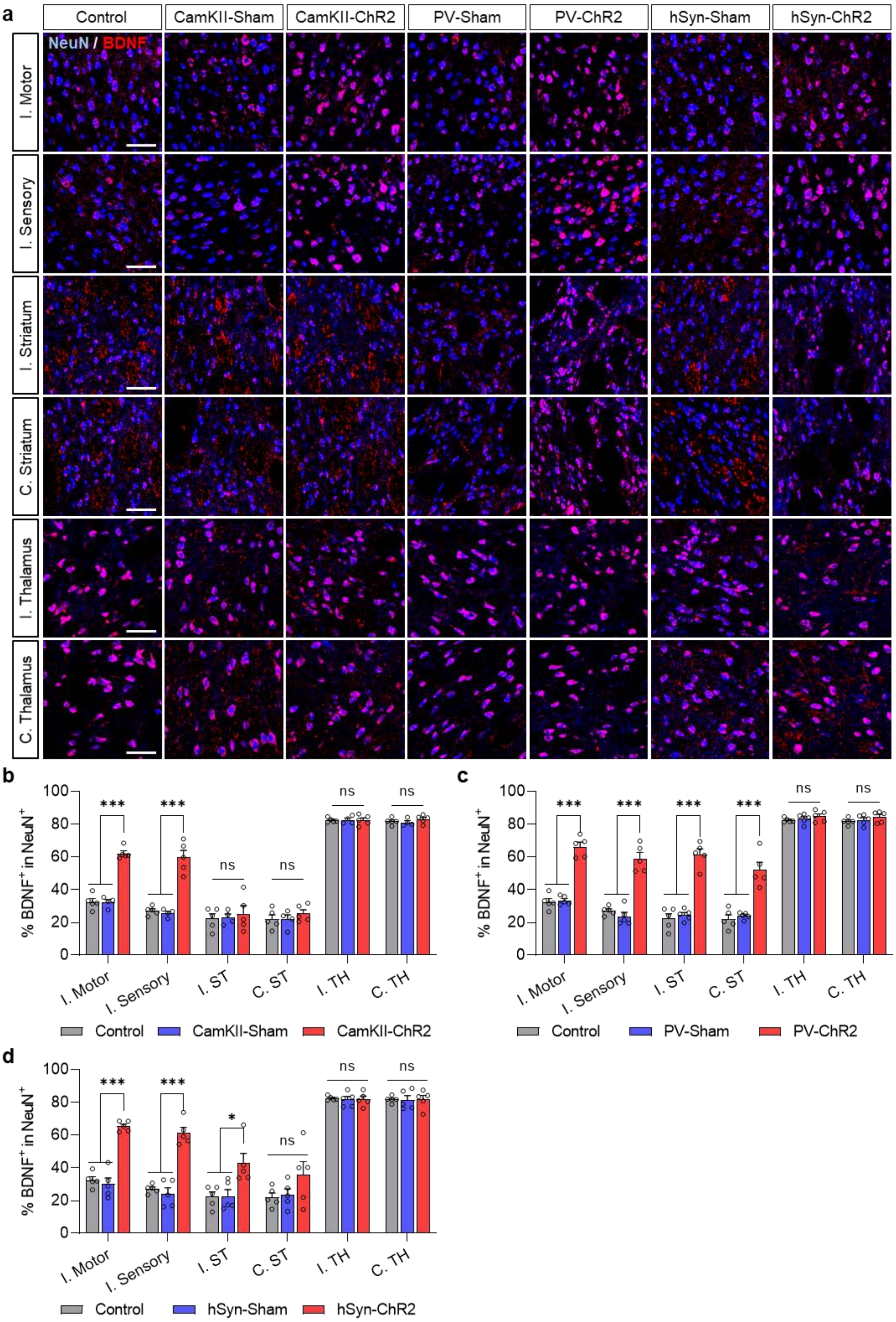

Supplementary Fig. 2. Patterns of BDNF expression for all experimental groups.

(a) Representative merged images of BDNF and NeuN stainings in six different ROIs for all experimental groups. **(b-d)** The percentage of $BDNF^+$ population in $NeuN^+$ cells in six different ROIs for all experimental groups (one-way ANOVA with Tukey`s multiple comparisons, *$p < 0.05$). All Data represent the mean ± S.E.M. *$p < 0.05$, **$p < 0.01$, ***$p < 0.001$. Scale bars: 50 µm.
